# FANCD2 genome binding is nonrandom and is enriched at large transcriptionally active neural genes prone to copy number variation

**DOI:** 10.1007/s10142-024-01453-5

**Published:** 2024-10-04

**Authors:** Justin L. Blaize, Jada Lauren N. Garzon, Niall G. Howlett

**Affiliations:** 1 Department of Cell and Molecular Biology, University of Rhode Island, 379 Center for Biotechnology and Life Sciences, 120 Flagg Road, Kingston, RI USA; 2 Present Address: Department of Chemical and Systems Biology, Stanford University School of Medicine, Stanford, CA 94305 USA

**Keywords:** Fanconi anemia, FANCD2, Genome instability, Mitotic DNA synthesis (MiDAS), Copy number variation (CNV)

## Abstract

Fanconi anemia (FA) is a rare genetic disease characterized by congenital abnormalities and increased risk for bone marrow failure and cancer. Central nervous system defects, including acute and irreversible loss of neurological function and white matter lesions with calcifications, have become increasingly recognized among FA patients, and are collectively referred to as Fanconi Anemia Neurological Syndrome or FANS. The molecular etiology of FANS is poorly understood. In this study, we have used a functional integrative genomics approach to further define the function of the FANCD2 protein and FA pathway. Combined analysis of new and existing FANCD2 ChIP-seq datasets demonstrates that FANCD2 binds nonrandomly throughout the genome with binding enriched at transcription start sites and in broad regions spanning protein-coding gene bodies. FANCD2 demonstrates a strong preference for large neural genes involved in neuronal differentiation, synapse function, and cell adhesion, with many of these genes implicated in neurodevelopmental and neuropsychiatric disorders. Furthermore, FANCD2 binds to regions of the genome that replicate late, undergo mitotic DNA synthesis (MiDAS) under conditions of replication stress, and are hotspots for copy number variation. Our analysis describes an important targeted role for FANCD2 and the FA pathway in the maintenance of large neural gene stability.

**Supplementary Information:**

The online version contains supplementary material available at 10.1007/s10142-024-01453-5.

## Introduction

Fanconi anemia (FA) is a rare genetic disease characterized by heterogeneous congenital abnormalities, increased risk for bone marrow failure (BMF) and cancer, accelerated aging, and premature mortality. The cumulative incidence of BMF among FA patients by age 40 years is 90%, and BMF can range from mild, asymptomatic cytopenias to severe aplastic anemia, myelodysplastic syndrome, or acute myelogenous leukemia (Shimamura and Alter 2010). FA patients are also at high risk for developing squamous cell carcinomas of the head, neck, and anogenital regions (Kutler et al. 2003). Therapeutic options for FA are extremely limited and the overall life expectancy of FA patients is approximately 29 years. The FA proteins are known to play a key role in DNA interstrand crosslink (ICL) repair in vitro (Kottemann and Smogorzewska 2013). An important role for the FA proteins in the detoxification of reactive aldehydes has also been established (Langevin et al. 2011; Langevin et al. 2013). Nevertheless, no rational therapeutic approaches based on the biochemistry of this disease have been developed.

In recent years, we have witnessed an increase in acute and chronic neurological symptoms in FA patients. These include limb weakness, papilledema, gait abnormalities,

headaches, dysphagia, visual changes, seizures, and progressive and irreversible loss of neurological function. These symptoms are accompanied by the appearance of numerous cerebral and cerebellar lesions with associated calcifications observed upon radiological imaging(Bartlett et al. 2024) (Eunike Velleuer-Carlberg, personal communication). For example, in a retrospective analysis of cranial MRI studies of 34 FA patients, at least one pathological brain imaging finding was observed in 22 (65%) patients. Of these 22 patients, six had mild to moderate intellectual disability, 3 had epilepsy, 1 had mild hearing loss, and 1 had hemiplegia (Aksu et al. 2020). In a similar study, brain structural abnormalities were observed in 18 (90%) of 20 FA patients, including pituitary and posterior fossa abnormalities, cerebellar atrophy, and morphological structural variation of the corpus callosum (Stivaros et al. 2015). Importantly, the molecular etiology of FANS is largely unknown.

In this study, we have employed both experimental and computational approaches to gain a greater understanding of the role of the FANCD2 protein in the maintenance of genome stability. Analysis of new and existing FANCD2 ChIP-seq datasets from both transformed and nontransformed cells clearly establishes that FANCD2 binds to the genome nonrandomly. Instead, FANCD2 genome binding is enriched at transcription start sites and in broad regions spanning protein-coding gene bodies, the latter referred to as broad binding regions (BBRs). These BBRs are enriched for large genes, many of which exceed 0.5 Mb in length. Network analysis of FANCD2 target genes across all datasets analyzed reveals an enrichment for neural genes encoding for proteins involved in differentiation, synapse function, and cell adhesion, and many of these genes have been linked to neurodevelopmental and neuropsychiatric disorders. Furthermore, FANCD2 BBRs overlap with regions of the genome that replicate late, undergo mitotic DNA synthesis (MiDAS) under conditions of replication stress, and are hotspots for copy number variation. Our analysis describes an important targeted role for FANCD2 and the FA pathway in the maintenance of large neural gene stability and offers potential insights into the molecular etiology of FANS.

## Materials and methods

### Cell culture and generation of cell lines

ACHT FA-D2 (*FANCD2*^*−/−*^) patient cells and the same cells complemented with wild-type FANCD2 were kindly provided by Detlev Schlinder at the University of Wuerzburg (Blaize et al. 2023). These cells harbor a maternally inherited missense hypomorphic mutation leading to an R815Q change and paternally inherited 2715 + 1G > A splice site variant that leads to a truncated protein. These cells were immortalized with human telomerase and cultured in Dulbecco’s Modified Eagle’s Medium (DMEM) supplemented with 18% (vol/vol) FBS, L-glutamine, penicillin-streptomycin, and 1 μg/ml puromycin. ACHT FA-D2 (*FANCD2*^*−/−*^) cells complemented with wild-type FANCD2 most likely express higher than normal physiological levels of FANCD2 in nontransformed cells.

### ChIP-seq

 ACHT FA-D2 (*FANCD2*^*−/−*^) cells complemented with wild-type FANCD2 were incubated in the absence or presence of aphidicolin (APH) for 24 h. Cells were harvested and washed with ice-cold PBS. Cell pellets were flash frozen and sent for ChIP-seq analysis with Active Motif Epigenetic Services. Chromatin immunoprecipitation was performed with rabbit polyclonal anti-FANCD2 antibody (NB100-182, Novus Biologicals). 75-nt single-end reads, at a read depth of 50 M reads per sample, were generated by standard Illumina sequencing on a NextSeq 500 sequencing platform. Reads were mapped to the human genome (hg19) using the Burrows Wheeler Aligner (bwa-v0.7.12) using default settings. Only reads that passed Illumina’s purity filter, aligned with no more than 2 mismatches, and mapped uniquely to the genome were used in the subsequent analysis. Duplicate PCR reads were removed. Aligned reads (tags) were extended to a length of 200 bp, corresponding to the average fragment length in the size-selected library. To determine the density of fragments (extended tags) along the genome, the genome was divided into 32-nt bins and the number of fragments in each bin determined. The following software was employed; bcl2fastq2 (v2.20), bwa (v0.7.12), Samtools (v0.1.19), BEDtools (v2.25.0), MACS2 (v2.1.0), SICER (v1.1), wigToBigWig (v4).

### Peak calling and merging

To determine significant peak enrichment over random background, peaks were called using MACS2 (*M*odel based *A*nalysis for *C*hIP-*S*eq) version 2.2.7.1, comparing treatment bam files to control input bam files using *P* value cut-offs of 1 × 10^− 4^ or 1 × 10^− 5^. Peak enrichment regions were generated using the bedtools merge option of the BEDTools suite version 2.30.0-GCC-10.2.0 with a maximum distance between peaks (-d) of 50,000 bp.

### Heatmap generation

deeptools_bam_compare/3.3.2.0.0 was used to normalize and compare sample and input bam files for each ChIP-seq dataset and to generate the log2 read ratio output as a bigwig file, using default settings. Data was prepared for heatmap generation using deeptools_compute_matrix/3.3.2.0.0 using the reference-point output option, with the reference point set as the transcription start site (TSS) with regions defined as 5,000 bp upstream and downstream of the TSS, using default settings. The region file used was UCSC Main on Human hg19 all UCSC genes May 2022. deeptools_plot_heatmap/3.3.2.0.1 was used to create heatmaps for score distributions across the specified region file.

### MiDAS analysis

To compare FANCD2 genome binding to genomic regions that undergo mitotic DNA synthesis (MiDAS), FANCD2 ChIP-seq peak enrichment regions were overlapped with MiDAS regions for U2OS, HeLa, and HS68 (Macheret et al. 2020). U2OS is a cell line with epithelial morphology derived from a moderately differentiated sarcoma of the tibia of a 15-year-old, White, female osteosarcoma patient. HeLa is a cervical carcinoma derived from a 31-year-old female patient. HS68 is a nontransformed fibroblast line isolated from the foreskin of an aspartoacylase deficiency White male patient. The coverage of MiDAS intervals/regions overlapping with FANCD2 ChIP-seq intervals/regions was determined using gops_coverage_1/1.0.0 on the Galaxy platform server (usegalaxy.org). For the MiDAS experiments, briefly, cells were synchronized with thymidine at the G1/S boundary and then released into S phase in the presence of aphidicolin (APH) and RO3306. APH slows down DNA replication, while RO3306, a CDK1 inhibitor, prevents entry into mitosis. Sixteen hours later, APH and RO3306 were removed, and EdU and nocodazole were added to the media. EdU labels nascent DNA, while nocodazole arrests cells in prometaphase and prevents mitotic exit. One hour later, the prometaphase-arrested cells were collected by mitotic shake-off and their EdU-labeled genomic DNA was affinity-purified and subjected to high throughput sequencing (Macheret et al. 2020). The MiDAS fastq sequencing data is available in the Sequence Read Archive (SRA) as BioProject PRJNA588267.

### CNV analysis

Bed files containing coordinates of *de novo* copy number variants were obtained from the Park et al. 2021 and Wilson et al. 2015 studies (Park et al. 2021; Wilson et al. 2015). HGMDFN090 (referred to as 090) cells are a normal, non-immortalized human skin fibroblast originally obtained from the Progeria Research Foundation (Peabody, MA) (Arlt et al. 2009). UMHF1 (referred to as HF1) is a non-transformed hTERT-immortalized human foreskin fibroblast line (Paulsen et al. 2013; Wilson et al. 2015). CNVs in 090 cells were detected using Illumina HumanOmni1 and HumanOmni2.5 BeadChip SNP microarrays and NimbleGen 12 × 270k array comparative genome hybridization (aCGH). *De novo* CNVs were detected in HF1 cells after cloning, using the HumanOmni2.5 BeadChip. The extent of overlap (coverage) between 090 and HF1 CNV regions overlapping with FANCD2 ChIP-seq regions was determined using gops_coverage_1/1.0.0 on the Galaxy platform server (usegalaxy.org).

### RNA-seq

 Cells were incubated in the absence or presence of aphidicolin (APH) for 24–48 h, harvested and washed with ice-cold PBS. Cell pellets were flash frozen and sent for sequencing with GENEWIZ/Azenta Life Sciences. RNA library prep was completed *via* polyA selection and HiSeq sequencing. RNA samples were quantified using Qubit 2.0 Fluorometer (Life Technologies, Carlsbad, CA) and RNA integrity was checked using Agilent TapeStation 4200 (Agilent Technologies, Palo Alto, CA). Sequencing libraries were prepared using NEBNext Ultra II RNA library prep kit using the manufacturer’s instructions (NEB, Ipswich, MA). Sequencing libraries were validated on the Agilent TapeStation and quantified using Qubit 2.0 Fluorometer as well as via quantitative PCR (KAPA Biosystems, Wilmington, MA). Sequencing libraries were clustered on a single lane of a flow cell and loaded onto an Illumina HiSeq 4000 instrument. Samples were sequenced using a 2 × 150 bp paired-end configuration at 60 M reads per sample. Reads were trimmed to remove adapter sequences and bases of poor quality using Trimmomatic v.0.36. Trimmed reads were then mapped to the hg19 ENSEMBL reference genome using STAR aligner v.2.5.2b. FeatureCounts subread package v.1.5.2 was run on BAM files generated from the alignment, to calculate reads uniquely mapping to genes in the genome. FeatureCounts output files were used for downstream differential expression analysis using DESeq2.

### Repli-seq analysis

FANCD2 ChIP-seq binding profiles were compared to replication timing profiles (Repli-seq) of IMR-90 normal lung fibroblasts obtained from the ENCODE Project Consortium repositories (ENCODE Project Consortium 2012; Luo et al. 2020); G1b phase (Accession number: ENCFF001GRA), G2 phase (ENCFF001GRD), S1 phase (ENCFF001GRG), S2 phase (ENCFF001GRA), S3 phase (ENCFF001GRM), and S4 phase (ENCFF001GRQ), from the Stamatoyannopoulos laboratory at the University of Washington. FANCD2 ChIP-seq binding profiles were also compared to the replication timing profiles of JEFF cells, an EBV-immortalized human B lymphocyte line, incubated in the absence or presence of 600 nM aphidicolin (APH) (Brison et al. 2019). Briefly, exponentially growing cells were pulse labeled with 50 μM BrdU, fixed in 70% ethanol, and incubated overnight at 4 °C in the presence of 15 μg/ml Hoescht 33,342. Cells were resuspended in phosphate-buffered saline and sorted based on their DNA content into six fractions by flow cytometry: G1 phase/very early S phase (G1/S1), S phases S2 through S5, and very late S, G2 and M phases (S6/G2/M). Cell fractions were lysed, DNA purified, and fragmented to a mean size of 500 bp. Fragmented DNA ends were repaired, and Illumina TruSeq indexed adapters were ligated. BrdU-labeled DNA was isolated by immunoprecipitation using an anti-BrdU monoclonal antibody, immunoprecipitated fragments were amplified, and the resulting libraries were purified. Illumina libraries were pooled and sequenced on a NextSeq 500 instrument on paired-end 2 × 43 or 2 × 75 bases, using a NextSeq 500/550 (Brison et al. 2019). Repli-seq bigWig files are available at NCBI GEO, accession number GSE134709.

## Results

### FANCD2 genome binding is non-random and enriched at transcription start sites in the absence or presence of DNA replication stress

**Fig. 1:**
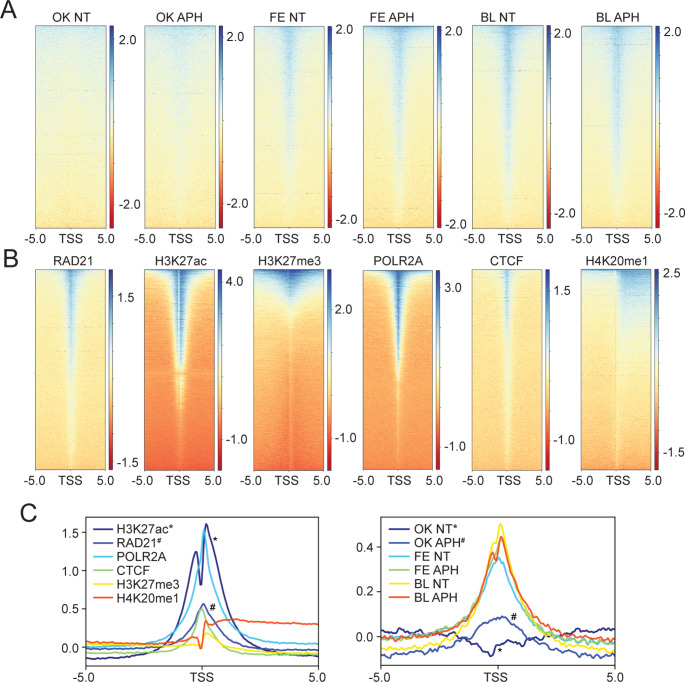
FANCD2 binding is non-random and enriched at transcription start sites. (**A**) Heatmaps of FANCD2 ChIP-seq data from U2OS (OK), HCT116 (FE), and FA-D2 + FANCD2 (BL) cells, before and after aphidicolin (APH) treatment. (**B**) Heatmaps from ENCODE ChIP-seq datasets of DNA binding proteins CTCF, POLR2A, and RAD21 and post-translationally modified histones H3K27ac, H3K27me3, and H4K20me1. Heatmaps were generated relative to the transcription start sites (TSS) (5,000 bp upstream and downstream) of UCSC genes and gene predictions from the GRCh37/hg19 genome assembly. (**C**) Read density plots of CTCF, POLR2A, RAD21, H3K27ac, H3K27me3, H4K20me1(left) and OK, FE and BL ChIP-seq data before and after (APH) treatment (right). Plots were generated relative to TSS. An asterisk (*) and hash (#) symbol are used to clearly distinguish the darker blue lines

In this study, we set out to analyze the genome-wide occupancy of the FANCD2 protein under unperturbed conditions and conditions of DNA replication stress, to determine if genome binding is random or nonrandom. FANCD2 genome occupancy under conditions of DNA replication stress has previously been examined in transformed cell models (Fernandes et al. 2021; Okamoto et al. 2018). Here, we chose to analyze FANCD2 genome binding in a non-transformed cell model, allowing us to compare patterns of binding between transformed and non-transformed models. We performed FANCD2 ChIP-seq in fibroblasts from an FA-D2 (*FANCD2*^*−/−*^) patient functionally complemented with FANCD2 (FA-D2 + FANCD2), cells recently characterized in our laboratory (Blaize et al. 2023). Cells were incubated in the absence (NT) or presence of aphidicolin (APH) for 24 h, and ChIP-seq was performed using a rabbit polyclonal anti-FANCD2 antibody. Heatmaps for FANCD2 genome occupancy in FA-D2 + FANCD2 (BL - this study), U2OS (OK) (Okamoto et al. 2018), and HCT116 (FE) (Fernandes et al. 2021) were generated relative to the transcription start sites (TSS) (5,000 bp upstream and downstream) of UCSC genes and gene predictions from the GRCh37/hg19 genome assembly (Fig. 1A). FANCD2 genome occupancy was compared to the genome occupancy of well-characterized DNA binding proteins (CTCF, POLR2A, RAD21) and post-translationally modified histones (H3K27ac, H3K27me3, H4K20me1) from publicly available ENCODE ChIP-seq datasets (ENCODE Project Consortium 2012). We observed a strong FANCD2 signal enrichment at transcription start sites (TSS) for all FANCD2 ChIP-seq datasets, except for the Okamoto non treated sample (Fig. 1A). FANCD2 enrichment at TSS closely resembled that for RAD21, a component of the cohesin regulatory complex, RNA Pol II (POLR2A), as well as CTCF, which specifies the locations of topologically associated domains (TADs) *via* the arrest of loop extrusion with the cohesin complex (Grubert et al. 2020) (Fig. 1B). In contrast, the binding profile of FANCD2 was distinct from that of H3K27ac, which exhibits a more diffuse enrichment at the TSS and active gene bodies, H4K20me1 enriched predominantly at active gene bodies, and H3K27me3 a transcriptionally repressive epigenetic mark. Read density plot profiles further illustrate the enrichment of FANCD2 at TSS, most closely resembling the profiles of RAD21, CTCF, and RNA Pol II (Fig. 1C). These data demonstrate that FANCD2 genome binding is nonrandom and is enriched at TSS in the absence or presence of DNA replication stress.

### FANCD2 genome binding is enriched at large genes

**Fig. 2:**
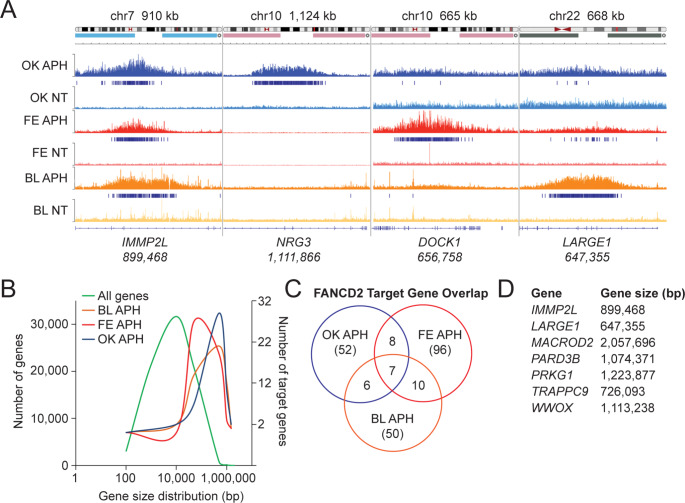
FANCD2 binds to large genes under conditions of replication stress. (**A**) Integrated Genomics Viewer (IGV) snapshot at *IMMP2L*, *NRG3*, *DOCK1*, and *LARGE1* genomic loci depicting shared and unique FANCD2 occupancy in OK, FE, and BL datasets following aphidicolin (APH) treatment. Specific genomic regions are displayed on top of the graphic. (**B**) Comparison of the gene size distribution of all genes in the human genome to the sizes of genes bound by FANCD2 in OK, FE, and BL datasets. (**C**) Venn diagram of unique and shared FANCD2 binding targets from OK, FE, and BL ChIP-seq datasets. (**D**) List of genes bound by FANCD2 in all three ChIP-seq datasets and their corresponding gene sizes

For all our subsequent analyses, we focus on FANCD2 genome binding following exposure to aphidicolin (APH), for reasons described below. Analysis of FANCD2 genome binding using the Integrated Genomics Viewer (IGV) (Robinson et al. 2011; Thorvaldsdóttir et al. 2013), revealed the clustering and enrichment of FANCD2 binding peaks across large genomic regions in all three datasets (Fig. 2A). We refer to these as FANCD2 broad binding regions (BBRs). FANCD2 BBRs frequently overlap large protein-coding genes, many exceeding 0.5 Mb in length (Fig. 2A). To quantify and rank these FANCD2 BBRs, we used the bedtools merge function to merge all peaks < 50 kb apart. For each dataset, merged peaks were ranked by their PSCORE (-log10 of cumulative peak *P* values) and a list of FANCD2 BBRs was generated for each dataset (Supplementary Tables S1-S3). Of the three datasets analyzed, the highest frequency of FANCD2 BBRs was observed for the Fernandes dataset (Fernandes et al. 2021). The overwhelming majority of BBRs overlapped protein coding genes; 91%, 82%, and 82% for Blaize, Fernandes, and Okamoto datasets, respectively. When comparing the size distribution of all genes in the genome to genes overlapping FANCD2 BBRs, we observed a strong skew toward large genes in all three datasets (Fig. 2B). For example, for the Blaize dataset we identified 53 BBRs. Of those 53 BBRs, 50 overlapped with a single gene, of which 13 and 34 genes were > 1.0 Mb and > 0.5 Mb, respectively. The other 3 BBRs either did not overlap with a gene or covered more than one gene (Supplementary Table S4). Similarly, for the Okamoto dataset, 58 of the 66 BBRs overlapped with a single gene and 16 and 46 of those BBR genes were > 1.0 Mb and > 0.5 Mb, respectively (Okamoto et al. 2018). A similar bias toward large genes was observed in the Fernandes dataset (Supplementary Table S4). In addition to a general strong binding bias for large genes, several of the same genes were bound by FANCD2 in more than one dataset (Fig. 2C). For example, 10 genes were bound by FANCD2 in both the Blaize and Fernandes datasets, while all three datasets shared 7 common binding genes, with all 7 genes > 0.5 Mb in length (Fig. 2C **and D**). Collectively, analysis of these datasets clearly establishes that FANCD2 genome binding under conditions of DNA replication stress is enriched across large genes in both transformed and nontransformed cells.

### Network analysis of FANCD2 target genes reveals an enrichment for neuronal processes and genes linked to neurodevelopmental and neuropsychiatric disorders

**Fig. 3:**
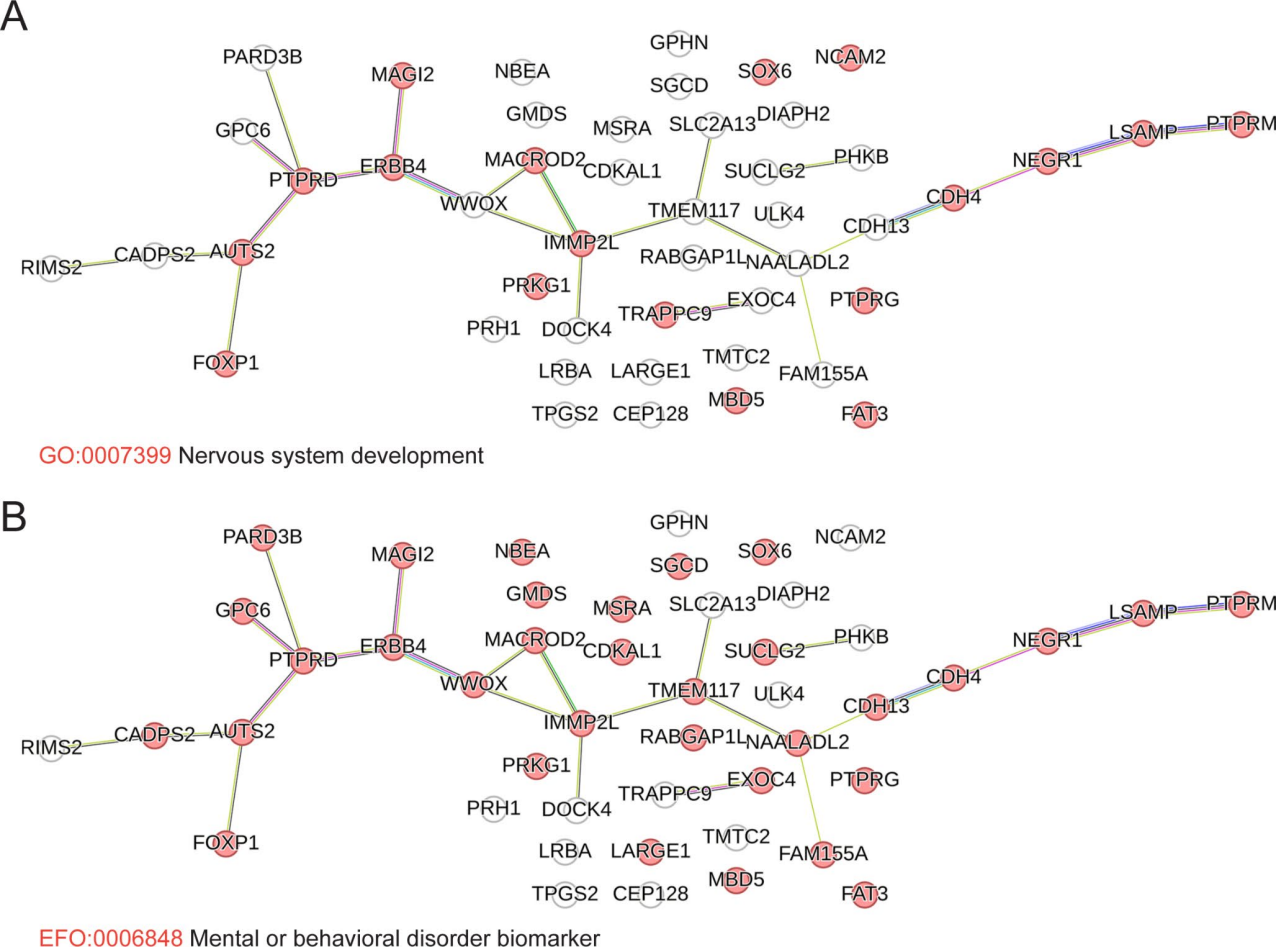
STRINGdb network analysis reveals FANCD2 binding enrichment at neural genes. (**A**) STRINGdb analysis of FANCD2 broad binding regions (BBRs) highlighting genes implicated in nervous system development in the BL ChIP-seq dataset. (**B**) STRINGdb analysis of FANCD2 BBRs highlighting genes associated with the human phenotype (Monarch) mental or behavioral disorder biomarker in the BL ChIP-seq dataset

STRING from the Swiss Institute of Bioinformatics is a database of known and predicted protein-protein interactions that stem from computational predictions, knowledge transfer between organisms, and interactions gathered from other primary databases (Snel et al. 2000). To determine if genes that FANCD2 binds to under conditions of replication stress have direct or predicted interactions and/or are associated with specific biological processes, we conducted network analysis using the STRING database. STRINGdb analysis of protein coding genes overlapping FANCD2 BBRs for all three datasets showed an enrichment for association with neuronal processes. Other detected enriched biological process gene ontologies included cell-cell adhesion, anatomical structure development, and cellular component organization. For neuronal process enrichment, 18 of 47 FANCD2 target genes from the Blaize dataset have an established or predicted role in nervous system development (FDR 0.0201) (Fig. 3A). Similarly, 24 of 48 FANCD2 target genes from the Okamoto dataset are associated with nervous system development (FDR 1.6 × 10^− 7^) (Supplementary Figure S1A). Many of these genes have also been linked to neurodevelopmental and neuropsychiatric disorders. For example, 33 of 47 FANCD2 target protein coding genes from the Blaize dataset are associated with the human phenotype (Monarch) mental or behavioral disorder biomarker (FDR 2.5 × 10^− 21^) (Fig. 3B). Similarly, 34 of 48 (FDR 2.9 × 10^− 22^) and 42 of 90 (FDR 6.8 × 10^− 18^) genes from Okamoto and Fernandes datasets, respectively, are linked to mental or behavioral disorders (Supplementary Figures S1B and C). Of note, FANCD2 was observed to bind to the *WWOX* gene in all three datasets. *WWOX* encodes for a short-chain oxidoreductase and is mutated in cerebellar ataxia and microcephaly syndrome patients with intellectual disability (Abdel-Salam et al. 2014; Mallaret et al. 2014). Similarly, FANCD2 bound to *TRAPPC9* in all three datasets (Fig. 2D). Mutations in the *TRAPPC9* gene are associated with autosomal recessive intellectual disability and mental retardation (Marangi et al. 2013; Mochida et al. 2009). In conclusion, network analysis of distinct FANCD2 ChIP-seq datasets demonstrates a common and striking enrichment for genes involved in neural processes and genetically linked to neurodevelopmental and neuropsychiatric disorders.

### FANCD2 BBRs overlap with genomic regions that undergo mitotic DNA synthesis under conditions of replication stress

**Fig. 4:**
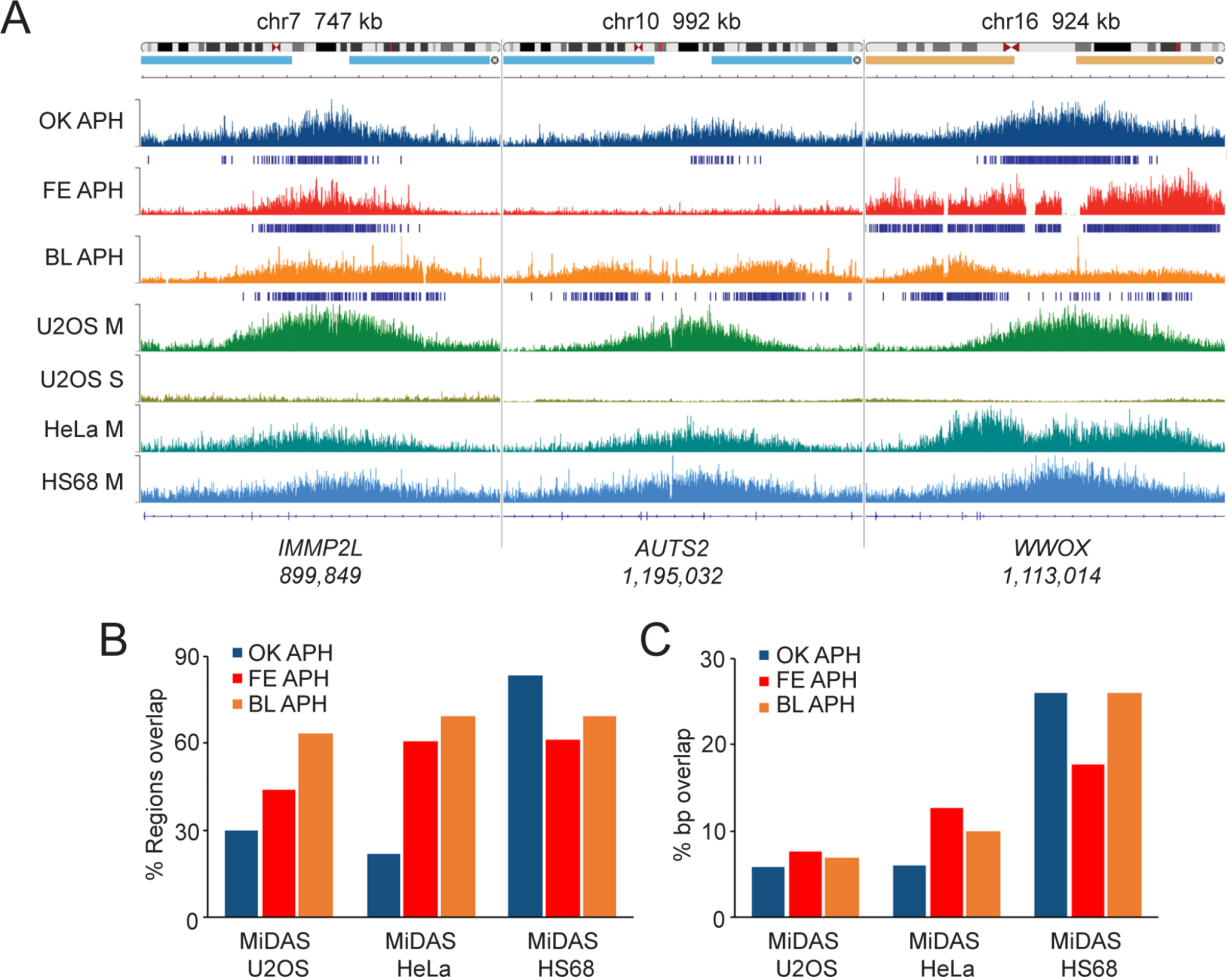
FANCD2 broad binding regions overlap with regions of the genome prone to mitotic DNA synthesis. (**A**) IGV snapshot of FANCD2 occupancy at the *IMMP2L*, *AUTS2*, and *WWOX* genes in OK, FE, and BL ChIP-seq datasets with the corresponding MiDAS-seq peaks from HeLa, U2OS, and HS68 cells at the same genomic loci from the Macheret et al. 2020 dataset (Macheret et al. 2020). Specific genomic regions are displayed on top of the graphic. (**B**) Bar graph depicting % region overlap between OK, FE, and BL FANCD2 ChIP-seq datasets and U2OS, HeLa, and HS68 MiDAS-seq data. (**C**) Bar graph depicting % base pair overlap between OK, FE, and BL FANCD2 ChIP-seq datasets and U2OS, HeLa, and HS68 MiDAS-seq data

The FANCD2 protein has been demonstrated to play an important role in mitotic DNA synthesis (MiDAS) - DNA replication that persists in M phase under conditions of DNA replication stress - in both cancerous and noncancerous cells (Graber-Feesl et al. 2019). Regions of the genome that undergo MiDAS have recently been mapped using a method known as mitotic DNA synthesis followed by high throughput sequencing (MiDAS-seq) (Ji et al. 2020; Macheret et al. 2020). Here, we sought to determine if regions of FANCD2 binding overlapped with MiDAS regions. Specifically, we compared FANCD2 genome binding in our three datasets to existing MiDAS datasets for two cancer-derived cell lines (HeLa and U2OS) and one non-cancer-derived line (HS68) (Macheret et al. 2020). Using IGV, we observed strong overlap between FANCD2 BBRs from all three datasets and MiDAS regions from HeLa, U2OS, and HS68 cells (Fig. 4A). For example, FANCD2 was observed to bind to the *IMMP2L* and *WWOX* genes in all three datasets, and the *AUTS2* gene in two of three datasets (Fig. 4A). All three genes show a strong MiDAS signal in U2OS, HeLa, and HS68 M phase samples, in contrast to U2OS S phase samples (Fig. 4A). Next, we calculated and plotted the degree of region and nucleotide overlap between FANCD2 ChIP-seq and MiDAS-seq datasets (Fig. 4B **and C**). We observed a high degree of region overlap between all three ChIP-seq and MiDAS-seq datasets, with the highest degree of overlap observed for HS68 MiDAS regions; 83%, 69%, and 61% of Okamoto, Blaize, and Fernandes BBRs overlapped with MiDAS regions from HS68 cells, respectively (Fig. 4B). Substantial region overlap was also observed with U2OS and HeLa MiDAS-seq regions, with 30%, 63%, and 44% of OK, BL, and FE BBRs overlapping with U2OS MiDAS regions, and 21%, 69%, and 61% of OK, BL, and FE BBRs overlapping with HeLa MiDAS regions, respectively (Fig. 4B). Base pair overlap analysis followed the same trend, with all three datasets having the highest overlap with HS68 MiDAS regions, followed by HeLa, and then U2OS (Fig. 4C). Exemplifying the late replicating properties of FANCD2 BBRs, comparison of our ChIP-seq data with Repli-seq replication timing profiles of IMR-90 cells, a normal lung fibroblast line, and JEFF cells, an EBV-immortalized human B lymphocyte line, demonstrates that FANCD2 BBRs overlap with regions that replicate late in the cell cycle, particularly under conditions of replication stress (Supplementary Figures S2A and B). Collectively, this analysis demonstrates that FANCD2 genome binding is enriched at regions of the genome that replicate late and undergo MiDAS under conditions of DNA replication stress.

### FANCD2 BBRs overlap with genomic hotspots for copy number variation

**Fig. 5:**
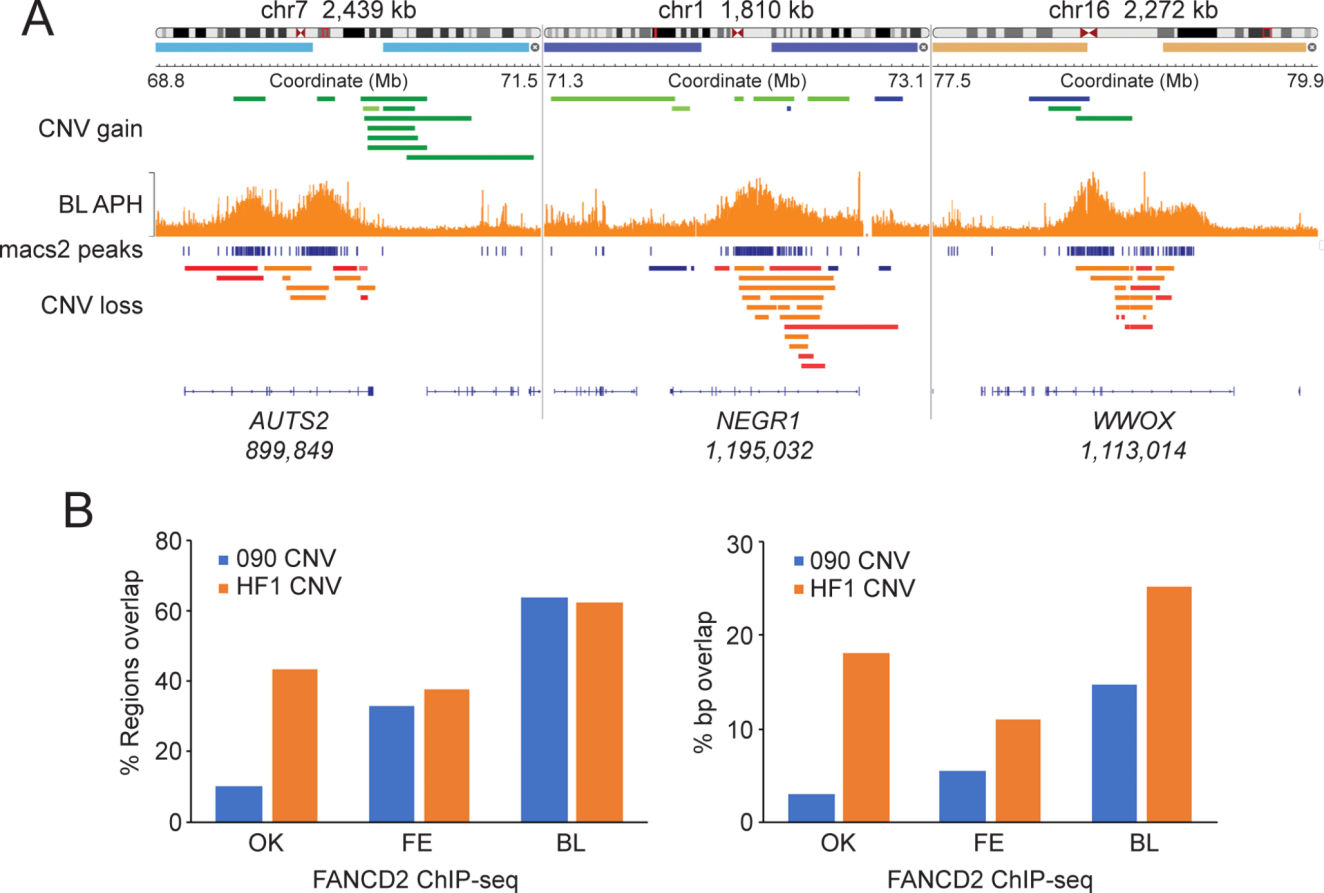
FANCD2 broad binding regions overlap with regions that are hotspots for copy number variation. (**A**) IGV snapshot of FANCD2 binding at the *AUTS2*, *NEGR1*, and *WWOX* genes with copy number variation gain (green) or loss (orange, red) data from the Wilson et al. 2015 study (Wilson et al. 2015) shown above and below the peak regions, respectively. Specific genomic regions are displayed on top of the graphic. (**B**) Bar graphs depicting % region overlap, and % base pair overlap between the OK, FE, and BL FANCD2 ChIP-seq datasets and copy number variation regions from 090 and HF1 cells from the Wilson et al. 2015 study (Wilson et al. 2015)

Our analysis thus far has shown that FANCD2 binds to large actively transcribed genes that replicate late and undergo MiDAS under conditions of replication stress (Fig. 2**and** Fig. 4). A common feature of large transcriptionally active and late replicating regions is that they are prone to genomic instability and copy number variation (CNV) (Wilson et al. 2015). CNVs include kb to Mb deletions and tandem duplications, inversions, as well as complex intrachromosomal rearrangements. To determine if FANCD2 BBRs are hotspots for CNV we compared the genome binding profiles of FANCD2 to that of recently reported CNVs from cultured human cell lines. Specifically, we compared CNV data - generated using SNP microarrays and array comparative genome hybridization (aCGH) - from two telomerase-immortalized normal human fibroblast lines (090 and HF1) to FANCD2 BBRs from all three ChIP-seq data sets (Wilson et al. 2015). We uncovered strong overlap between FANCD2 BBRs and CNV hotspots as demonstrated in IGV (Fig. 5A). For example, the *AUTS2*, *NEGR1*, and *WWOX* loci are hotspots for CNV gains and losses in both 090 and HF1 and regions of strong FANCD2 binding in the Blaize FANCD2 ChIP-seq dataset (Fig. 5A). FANCD2 binding was detected at *AUTS2* and *NEGR1* in two of the three ChIP-seq datasets while FANCD2 binding to *WWOX* was detected in all three datasets (Supplementary Tables S1-S3). We also performed coverage analysis to quantify the extent of overlap between CNV hotspots and FANCD2 BBRs for all three datasets, confirming a high degree of concordance between CNV and FANCD2 ChIP-seq regions (Fig. 5A **and B**). FANCD2 BBRs from the Blaize dataset showed the highest degree of CNV overlap (regions and bp) with 63% and 62% of BBRs overlapping with CNV hotspots in 090 and HF1 cells, respectively (Fig. 5B). Considerable overlap was also uncovered for both the Fernandes and Okamoto BBRs. For example, 33% and 37% of Fernandes regions overlapped with CNV hotspots in 090 and HF1 cells, respectively (Fig. 5B). These findings reveal that FANCD2 binds to regions in the genome that are prone to structural variation under conditions of replication stress.

### Loss of FANCD2 impacts gene expression of FANCD2 BBR target genes

**Fig. 6:**
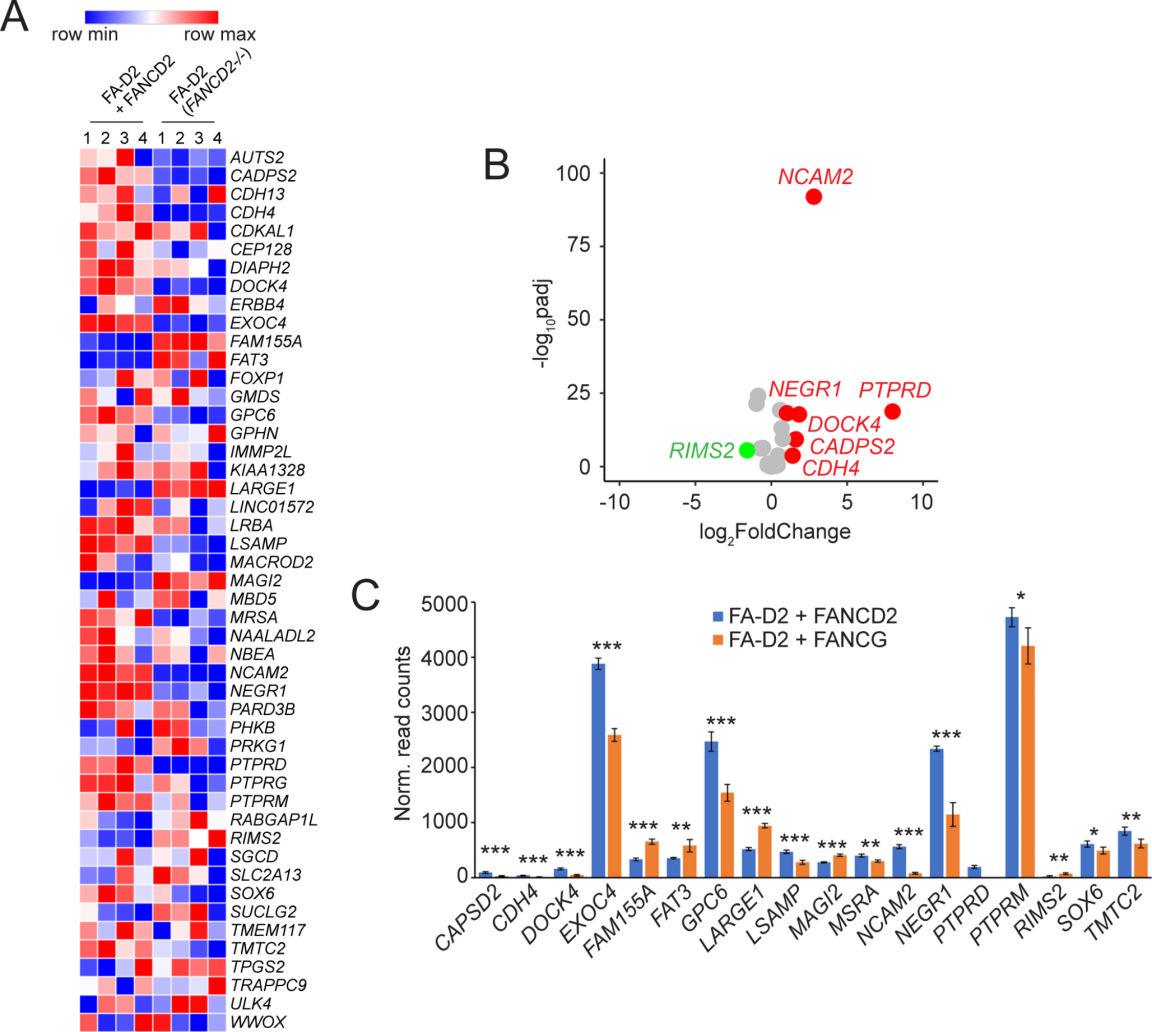
Loss of FANCD2 impacts gene expression of FANCD2 target genes. (**A**) Morpheus heatmap analysis of RNA-seq data demonstrating differential expression of FANCD2 broad binding region (BBR) genes in FA-D2 (*FANCD2*^*−/−*^) cells relative to FA-D2 + FANCD2 cells. Genes with increased expression in FA-D2 (*FANCD2*^*−/−*^) cells relative to FANCD2-complemented FA-D2 cells are depicted in red and genes with decreased expression are depicted in blue. (**B**) Log2 fold change expression of FANCD2 BBR genes between FA-D2 + FANCD2 and FA-D2 (*FANCD2*^*−/−*^) cells after APH treatment. Genes upregulated in FA-D2 + FANCD2 cells - with an adjusted *P* value less than 0.05 and a log2 fold change greater than 1 - are shown in red. Genes downregulated in FA-D2 + FANCD2 cells - with an adjusted *P* value less than 0.05 and a log2 fold change less than − 1 - are shown in green. (**C**) Normalized read counts of FANCD2 BBR genes after APH treatment. FA-D2 + FANCD2 read counts are shown in blue and FA-D2 (*FANCD2*^*−/−*^) are shown in orange. *, *P* < 0.05; **, *P* < 0.01; ***, *P* < 0.001

Our combined ChIP-seq analysis establishes that FANCD2 preferentially binds to protein-coding genes under conditions of DNA replication stress (Fig. 2). For example, all but one of 53 BBRs in the FA-D2 + FANCD2 cells mapped to gene bodies. To determine if loss of FANCD2 influences levels of expression of these genes, we performed RNA-seq analysis on our non-transformed FA-D2 (*FANCD2*^*−/−*^) and FA-D2 + FANCD2 cells incubated in the absence or presence of DNA replication stress. Of the 50 protein coding genes that FANCD2 binds to, we detected expression of 48 of these genes in both FA-D2 (*FANCD2*^*−/−*^) and FA-D2 + FANCD2 cells (Fig. 6and Supplementary Table S5). This is consistent with previous ChIP-seq studies demonstrating that FANCD2 binds preferentially to transcriptionally active genes (Okamoto et al. 2018). Morpheus heatmap analysis of these genes revealed a general pattern of differential gene expression between FA-D2 (*FANCD2*^*−/−*^) and FA-D2 + FANCD2 cells (Fig. 6A). Of these 48 expressed genes, 7 exhibited a log2 fold change greater than 1 or less than − 1 (Fig. 6B). Furthermore, 18 of these genes exhibited a statistically significant difference in levels of gene expression (Fig. 6C). For those differentially expressed genes, we did not observe any correlation between gene length, numbers of exons, expression levels, or transcript/mRNA length, and their propensity for differential expression (Supplementary Table S5). Our results indicate that genes bound by FANCD2 under conditions of replication stress are transcriptionally active and that loss of FANCD2 can impact the levels of expression of these genes.

## Discussion

In vitro studies have established a major role for the FA pathway in the repair of DNA interstrand crosslinks (Kottemann and Smogorzewska 2013). Much less is known about the endogenous source of genome instability characteristic of FA patient cells. We reasoned that clues to this physiological function might be gleaned from determining if the FA pathway exhibits specificity for the repair of particular genomic regions. To explore this question, we employed experimental and comparative genomics approaches to gain greater insight into the genomic context of FANCD2’s DNA repair activity. Concurrent and parallel analysis of new and existing FANCD2 ChIP-seq datasets, from both transformed and nontransformed cells, clearly establishes that FANCD2 binds to the genome in a nonrandom manner. FANCD2 was found to be enriched at transcription start sites and in broad binding regions (BBRs) spanning protein-coding genes. Notably, these BBRs are enriched for large genes, many of which exceed 0.5 Mb in length. BBRs overlap with regions of the genome that replicate late, undergo mitotic DNA synthesis (MiDAS) under conditions of replication stress, and are hotspots for copy number variation. For the ChIP-seq analysis performed in this study, accompanying RNA-seq analysis demonstrated that the majority of genes bound by FANCD2 are transcriptionally active. A similar correlation between transcriptional activity and FANCD2 binding has been previously observed (Okamoto et al. 2018). In this study, the accumulation of FANCD2 at large transcriptionally active genes was partially attributed to the formation of R-loops - RNA-DNA hybrids with a displaced strand of DNA - most likely associated with transcription-replication conflicts. The accumulation of FANCD2 at select loci was decreased by treatment with cordycepin, a transcription inhibitor, and by treatment with RNaseHI, which hydrolyzes the RNA component of R-loops (Okamoto et al. 2018). An important role for the FANCD2 protein in the resolution of R-loops under conditions of replication stress has previously been established (Garcia-Rubio et al. 2015; Liang et al. 2019).

Here, we have also established that FANCD2 localizes to regions of the genome prone to genome instability in the form of copy number variation (CNV). CNVs are kb to Mb gains and losses and are both a source of normal genetic variation and a major cause of neurodevelopmental and neuropsychiatric disorders, including autism spectrum disorder and schizophrenia (Conrad et al. 2009). The Cancer Genome Atlas (TCGA) and other studies have shown that CNVs arise frequently in cancers and that certain genomic loci, including tumor suppressor genes and common chromosomal fragile sites (CFSs), are highly prone to their occurrence (Beroukhim et al. 2010; Hoadley et al. 2014; Lupski 2007; Pleasance et al. 2010). The environmental factors and cellular mechanisms contributing to the instability of these loci is thus of broad importance. We previously established an important role for the FANCD2 protein and the FA pathway in the maintenance of CFS stability under conditions of replication stress (Howlett et al. 2005; Madireddy et al. 2016). The results of this study expand on these findings and suggest that FANCD2 may play a broader role in the stabilization of genome-wide loci prone to CNV, and experiments are ongoing to directly test this hypothesis. The highest degree of FANCD2 genome binding and CNV overlap was observed for nontransformed FA-D2 (*FANCD2*^*−/−*^) cells complemented with wild-type FANCD2. This may be reflective of similar transcriptional profiles between FA-D2 + FANCD2, 090, and HF1 cells, which are all fibroblast lines, with transcriptional activity being closely associated with FANCD2 binding and propensity for CNV formation (Blaize et al. 2023; Okamoto et al. 2018; Wilson et al. 2015). Nevertheless, FANCD2 genome binding and CNV overlap was observed for both nontransformed and transformed lines, suggesting a broader role for FANCD2 in the stabilization of genome-wide loci prone to CNV.

While these studies suggest an important role for FANCD2 in the prevention of CNV under conditions of replication stress, the mechanisms by which FANCD2 might prevent CNV formation is unclear. The role of FANCD2 in the removal and/or suppression of R-loops might be central to this function (Garcia-Rubio et al. 2015; Liang et al. 2019). However, in the Okamoto et al. 2018 study, the role of R-loop formation in FANCD2 genome binding was assessed solely at a single gene locus, *NRG3* (Okamoto et al. 2018). Similarly, a previous study postulated that enhanced R-loop formation is a major contributor to common chromosomal fragile site instability, albeit analysis was limited to the *FHIT* gene at FRA3B (Helmrich et al. 2011). In a recent study by Park et al. 2021; examining the mechanisms of CNV formation and large gene instability, analysis of genome-wide DRIP-seq (DNA: RNA hybrid immunoprecipitation followed by next-generation sequencing) datasets revealed a low burden of R-loops at large transcribed genes (Park et al. 2021). Furthermore, in the same study, the knockdown or overexpression of RNase H1 did not have a significant impact on replication stress-induced CNV formation or CFS instability (Park et al. 2021). Here the authors speculate that the role of R-loop formation in large transcriptionally active gene instability might be secondary to fork failure occurring for several other reasons. Multiple causative mechanisms for large gene instability have been proposed including the following; long-traveling replication forks moving through replication origin-poor regions; the transcription of large genes extending beyond a single cell cycle and leading to transcription and replication machinery collisions (Helmrich et al. 2011); and/or the uncoupling of the replicative helicases and polymerases, all of which would be exacerbated under conditions of replication stress (Arlt et al. 2012; Kaushal and Freudenreich 2019).

Our analysis also demonstrates a strong overlap between FANCD2 binding regions and regions of the genome that frequently undergo mitotic DNA synthesis (MiDAS), a replicative mechanism activated to resolve unreplicated DNA prior to anaphase in mitosis (Minocherhomji et al. 2015; Özer and Hickson 2018). An important role for FANCD2 and RAD52 in MiDAS has previously been established (Bhowmick et al. 2016, 2023; Graber-Feesl et al. 2019). Intriguingly, while RAD52 is dispensable for MiDAS in nontransformed cells, FANCD2 remains indispensable (Graber-Feesl et al. 2019). Other HR proteins such as BRCA2 and RAD51 are also dispensable for this process (Bhowmick et al. 2023; Feng and Jasin 2017), suggesting that MiDAS and/or the resolution of MiDAS intermediates is not strictly a recombination-mediated process and is uniquely dependent on FANCD2. Deciphering the mechanistic role of FANCD2 in this process is likely to greatly improve our understanding of the physiological role of FANCD2 and the FA pathway and provide further avenues of investigation for much needed improved therapeutic approaches for this disease.

## Electronic supplementary material

Below is the link to the electronic supplementary material.

Supplementary Material 1

Supplementary Material 2

Supplementary Material 3

Supplementary Material 4

Supplementary Material 5

Supplementary Material 6

Supplementary Material 7

## Data Availability

We are in the process of depositing our data to the European Nucleotide Archive.
