# Supplementary material for: FANCD2 genome binding is nonrandom and is enriched at large transcriptionally active neural genes prone to copy number variation
Source: Funct Integr Genomics. 2024 Oct 4;24(5):180. doi: 10.1007/s10142-024-01453-5 (PMC11452531; doi:10.1007/s10142-024-01453-5)
Supplement: Supplementary file 6 — Supplementary Material 6 [file 10142_2024_1453_MOESM6_ESM.docx]

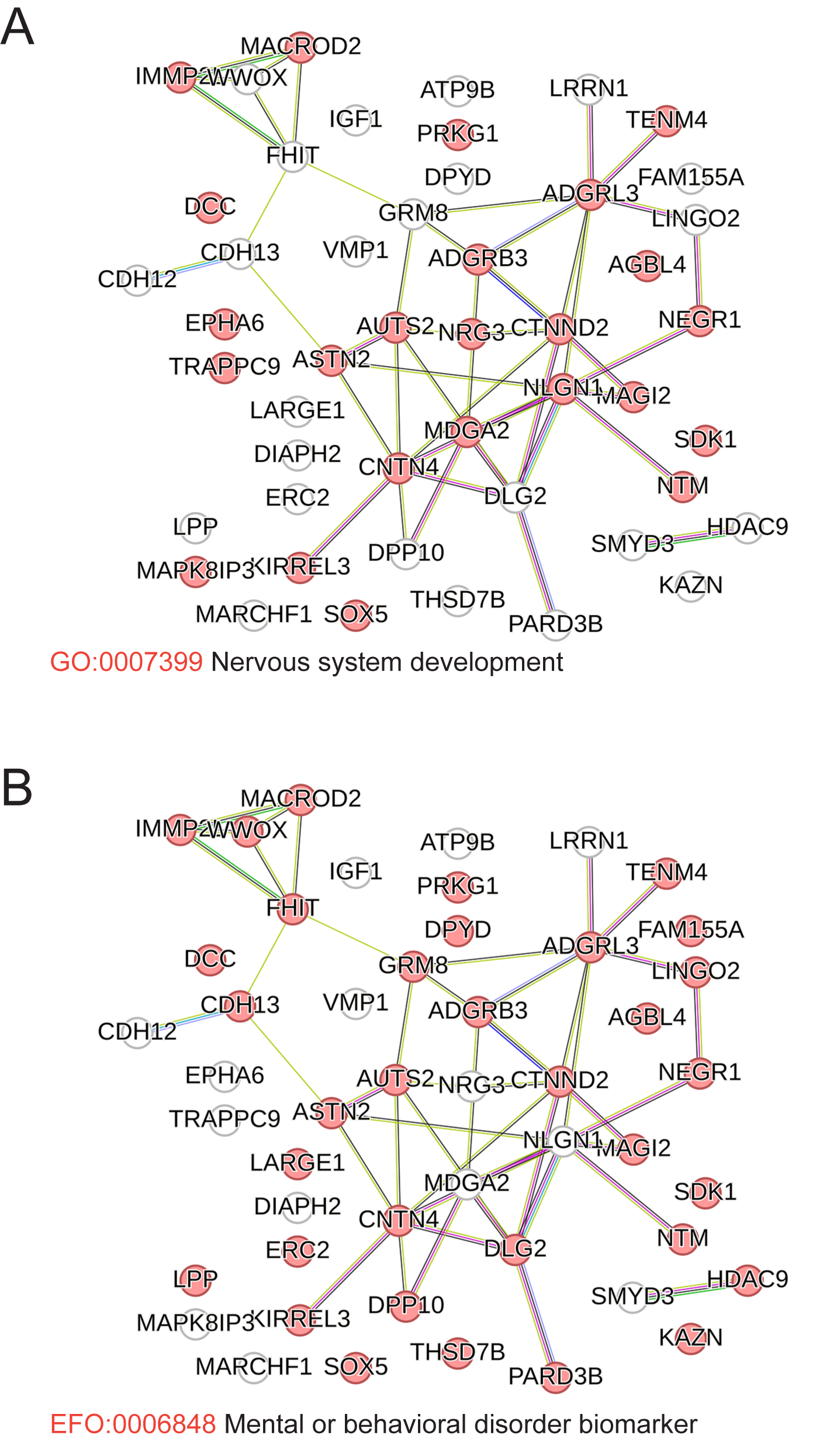


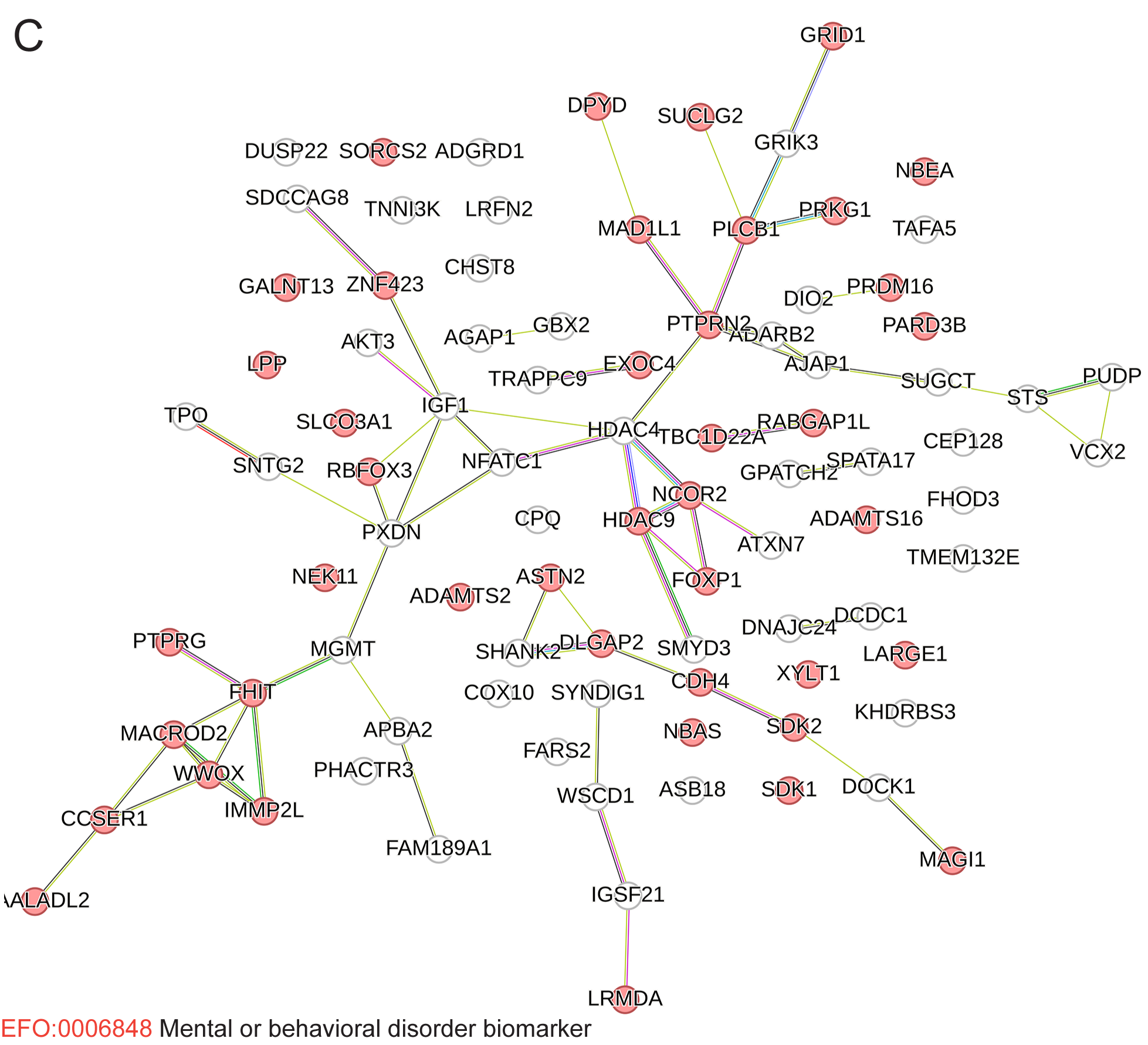


**Figure S1. STRINGdb network analysis reveals FANCD2 binding enrichment at neural genes in U2OS and HCT116 cells.**  (A) STRINGdb network analysis of FANCD2 broad binding regions (BBRs) highlighting genes involved in nervous system development in the OK ChIP-seq dataset. (B) STRINGdb network analysis of FANCD2 BBRs highlighting genes associated with the human phenotype (Monarch) mental or behavioral disorder biomarker in the OK ChIP-seq dataset. (C) STRINGdb network analysis of FANCD2 BBRs highlighting genes associated with the human phenotype (Monarch) mental or behavioral disorder biomarker in the FE ChIP-seq dataset.
