# Supplementary material for: FANCD2 genome binding is nonrandom and is enriched at large transcriptionally active neural genes prone to copy number variation
Source: Funct Integr Genomics. 2024 Oct 4;24(5):180. doi: 10.1007/s10142-024-01453-5 (PMC11452531; doi:10.1007/s10142-024-01453-5)
Supplement: Supplementary file 7 — Supplementary Material 7 [file 10142_2024_1453_MOESM7_ESM.docx]

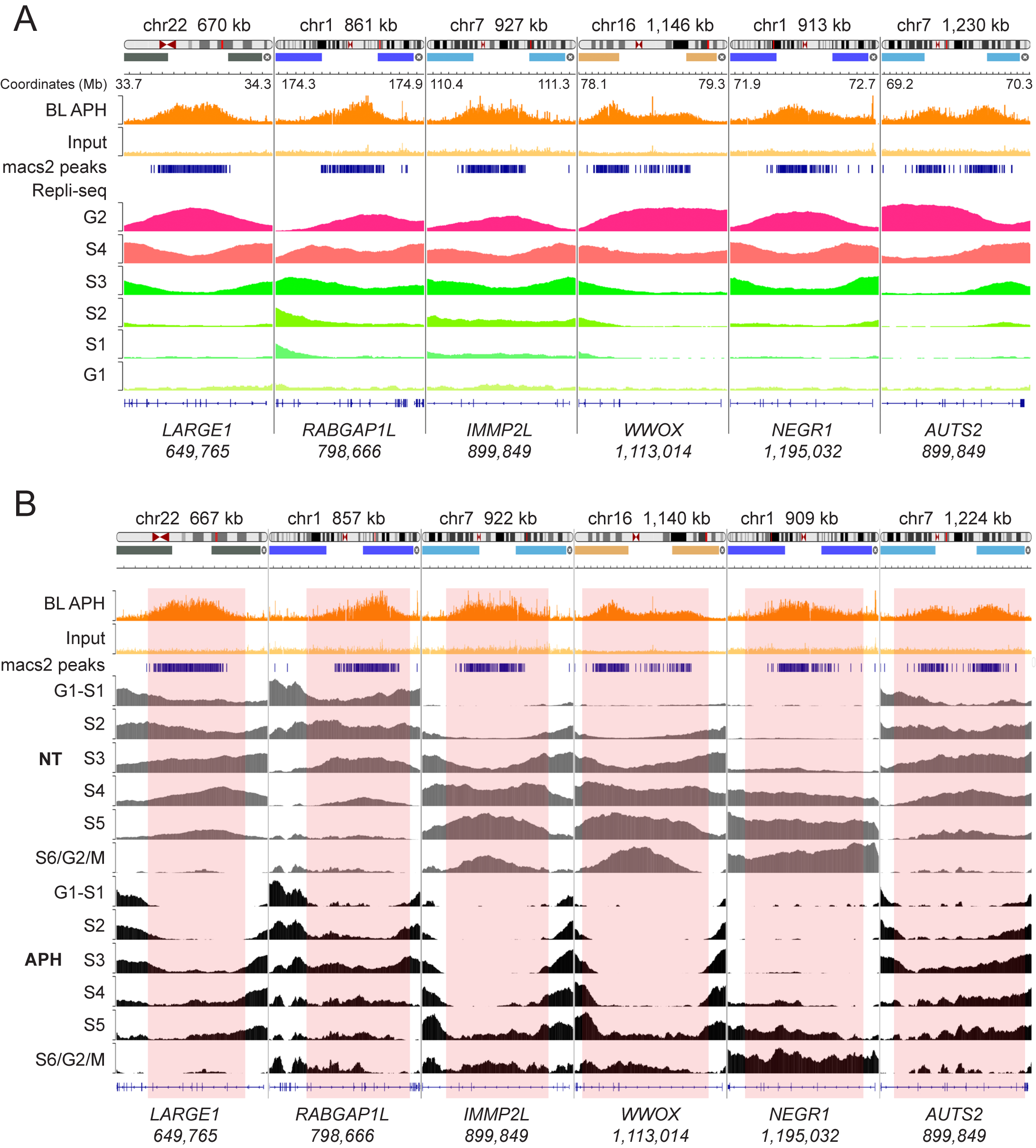


**Figure S2. FANCD2 binding is enriched at late replicating regions of the genome.** (A) Repli-seq replication timing profiles of IMR-90 cells at the *LARGE1*, *RABGAP1L*, *IMMP2L*, *WWOX*, *NEGR1*, and *AUTS2* genes under non-treated conditions. FANCD2 occupancy from the BL ChIP-seq dataset is shown for all genes. Specific genomic regions are displayed on top of the graphic. (B) Repli-seq replication timing profiles of JEFF cells at the *LARGE1*, *RABGAP1L*, *IMMP2L*, *WWOX*, *NEGR1*, and *AUTS2* before and after APH treatment. FANCD2 occupancy from BL ChIP-seq dataset is shown for all genes. Specific genomic regions are displayed on top of the graphic.
